# Elevated Autotaxin and LPA Levels during Chronic Viral Hepatitis and Hepatocellular Carcinoma Associate with Systemic Immune Activation

**DOI:** 10.3390/cancers11121867

**Published:** 2019-11-25

**Authors:** Lenche Kostadinova, Carey L Shive, Donald D Anthony

**Affiliations:** 1 Department of Medicine, University Hospitals Medical Center, and the Center for AIDS Research, Case Western Reserve University, Cleveland, OH 44106, USA; lxk194@case.edu; 2 The Louis Stokes VA Medical Center, Cleveland, OH 44106, USA; carey.shive@case.edu; 3 Department of Pathology, Case Western Reserve University, Cleveland, OH 44106, USA; 4 Department of Medicine, Division of Rheumatology, MetroHealth Medical Center, Case Western Reserve University, Cleveland, OH 44109, USA

**Keywords:** autotaxin, Lysophosphatidic acid, Hepatitis, liver, Hepatocellular Carcinoma, inflammation, immune activation, immunity, aging

## Abstract

Circulating autotaxin (ATX) is elevated in persons with liver disease, particularly in the setting of chronic hepatitis C virus (HCV) and HCV/HIV infection. It is thought that plasma ATX levels are, in part, attributable to impaired liver clearance that is secondary to fibrotic liver disease. In a discovery data set, we identified plasma ATX to be associated with parameters of systemic immune activation during chronic HCV and HCV/HIV infection. We and others have observed a partial normalization of ATX levels within months of starting interferon-free direct-acting antiviral (DAA) HCV therapy, consistent with a non-fibrotic liver disease contribution to elevated ATX levels, or HCV-mediated hepatocyte activation. Relationships between ATX, lysophosphatidic acid (LPA) and parameters of systemic immune activation will be discussed in the context of HCV infection, age, immune health, liver health, and hepatocellular carcinoma (HCC).

## 1. Introduction

Autotaxin(ATX) is a member of the family of nucleotide pyrophosphatases/phosphodiesterases (NPPs) [1]. Expression of autotaxin is ubiquitous, with relatively high levels in adipose tissue, brain, kidney, and lymphoid organs [2], including the high endothelial venule, where a role in lymphocyte trafficking has been proposed [3]. ATX has lysophospholipase D activity, and it is the major enzyme catalyzing the formation of lysophosphatidic acid (LPA) in the blood [4,5]. The ATX-LPA axis has been studied in airway inflammation and wound healing, as well as a variety of cancers such as breast, ovarian, lung, colorectal, melanoma, pancreatic, thyroid, liver, and glioblastoma multiforme [6,7,8,9,10,11,12,13,14,15]. At the level of immune modulation, the ATX-LPA axis is a strong inducer of inflammatory mediators like IL-8, IL-6, TNFα, and growth factors such as the vascular endothelial growth factor (VEGF) and granulocyte colony-stimulating factor (G-CSF) [16]. When acute inflammation becomes chronic, ATX-LPA signaling contributes to augmented cytokine production and lymphocyte infiltration, aggravating inflammation in conditions such as asthma, pulmonary fibrosis, rheumatoid arthritis, and liver fibrosis [16,17,18,19,20,21]. Here we will review how ATX-LPA homeostasis is altered during chronic hepatitis C virus (HCV) and HCV/HIV infection, how this may contribute to systemic immune activation, and how the ATX-LPA axis is related to liver cancer.

## 2. Autotaxin Is Elevated during Liver Disease, Including during Chronic HCV Infection

Elevated serum/plasma levels of ATX have been well described in patients with chronic liver disease [22,23,24,25], including cholestatic liver disease, both primary biliary cirrhosis (PBC) and primary sclerosing cholangitis (PSC). Elevated ATX levels are associated with pruritus, severity of liver injury, disease progression and overall survival [24,25]. Additionally, ATX levels are elevated in chronic hepatitis B virus (HBV) infection, HCV infection, non-alcoholic fatty liver disease (NAFLD) and levels are associated with the degree of liver fibrosis (Table 1) [22,23,26,27,28,29]. 

In normal liver, ATX is taken up from the hepatic sinusoids and degraded by liver sinusoidal endothelial cells (LSEC). In fact ^125^I labeled ATX when injected into the mouse tail vein accumulates in the liver within 5 minutes in a scavenger receptor mediated manner [34], indicating the liver plays a role in ATX homeostasis. In fibrotic liver tissue, capillarization of the sinusoids occurs. This is accompanied by impairment of ATX uptake. In this way, liver fibrosis is thought to contribute to reduced ATX clearance and to elevated ATX plasma levels [18,34,35]. Thus, it is thought that any liver disease that leads to fibrosis is capable of contributing to elevated ATX levels. It has been shown that ATX can be produced locally and specifically by hepatocytes upon toxin (carbon tetracholoride )-induced chronic liver damage [36]; TNF additionally can stimulate ATX expression in hepatoma cells in vitro through nuclear factor kappa B (NF-kB) signaling [37]. Notably, adipose tissue expresses what is thought to be the majority of circulating ATX, and conditional gene knockout of ATX in adipose tissue in mice results in up to a 38% decrease in circulating LPA levels demonstrating the importance of adipose tissue in ATX homeostasis [38]. In comprehensive stable isotope labeling studies (deuterium labeled H2O), the turnover rate (degradation and production rate, or half-life) of ATX at equilibrium in a healthy young human is thought to be approximately 2.3 days [39], suggesting that in the non-disease state there is a relatively rapid (albeit slower than a time frame of hours) turnover of this protein. While the contributors to ATX homeostasis during the disease state are not well understood, it appears that adipose tissue and the liver play some role in production and some component of impaired liver function affects its clearance.

As shown in the Table 1 there are at least 3 main methods used to measure ATX in serum and plasma. One is a two-site immunoassay that measures ATX concentration using an automated analyzer. The ATX assay reagent consist of beads coated with the F(ab)_2_ fragment of a ATX specific primary antibody combined with a second ATX specific antibody that is conjugated to alkaline phosphatase. When the ATX assay reagent is mixed with buffer and human serum the assay reaction is quick and yields automated, high throughput results [40]. This assay correlates well with lysophospholipase D activity in human serum. Another method of analyzing serum/plasma ATX is via an activity assay that determines the amount of liberated choline by enzymatic fluorimetry [41]. Finally, ATX can be measured in serum/plasma by using enzyme-linked immunosorbent assay employing enzyme-linked polyclonal antibody specific for human ATX. 

Hepatocellular carcinoma (HCC) often develops in patients with chronic liver injury and liver fibrosis [42]. Patients with HCC have elevated serum levels of ATX when compared with healthy controls. It is not clear whether this is attributable to the cancer or to liver fibrosis associated with cancer [32,33,40]. In at least one report examining serum levels of ATX in patients with liver cirrhosis, no difference was seen between patients with HCC compared to those without HCC [32], and there was no difference in serum ATX levels before or after radiofrequency ablation of the tumor [32]. When liver tissue was examined for ATX mRNA, it was overexpressed in patients with HCC compared to normal liver tissue [37,43,44]. Expression was found in 89% of tumor tissues, especially in patients with cirrhosis or HCV infection, compared to 20% in normal hepatocytes [37]. Furthermore, ATX mRNA expression in liver tissue of HCC patients was correlated with inflammation, as measured by serum aspartate aminotransferase (AST)and alanine aminotransferase (ALT), and with liver cirrhosis [37]. Additionally, in patients with HCC high levels of liver ATX mRNA, vascular invasion and poor tumor differentiation was more frequent [33,45]. Notably, one study indicates ATX mRNA expression is not elevated in HCC tissue, but rather lower compared to the background liver tissues, and there was no correlation between serum ATX level and ATX mRNA expression in HCC patients [32]. It therefore, appears unclear whether the HCC disease state, liver fibrosis, or a combination of the two contribute to elevated tissue ATX mRNA expression and serum/plasma ATX levels in patients with combinations of liver disease, liver fibrosis, and HCC.

## 3. Elevated Plasma LPA Levels Are Present during HCV Infection and Likely HCC

The enzymatic byproduct of ATX activity is Lysophosphatidic Acid (LPA), and in general, elevated ATX serum levels correlate with elevated LPA levels [4,46]. There is consensus that plasma LPA levels are elevated during HCV infection. Watanabe et al. have shown elevated plasma LPA levels measured by colorimetric assays using an enzymatic cycling method in chronic HCV infection. We have also shown LPA 16:0 and LPA 20:4 subtype levels to be elevated in a small cohort of subjects with HCV compared to uninfected, age range matched controls [23]. Regarding HCC, one study has shown serum/plasma LPA levels may be greater in patients with HCC than patients with liver cirrhosis and no HCC [47]. Additionally, a study using plasma mass spectroscopy analysis indicated higher plasma levels of the LPA 20:4 subtype in patients with HCC compared to patients without HCC and higher plasma levels of the LPA 18:2 subtype in patients with HCV infection compared to patients with HCV and HCC [48]. Overall, there is consensus that plasma LPA levels are elevated during HCV infection; though which specific subtypes are elevated is less clear. It also appears that serum LPA levels are altered in the setting of HCC, though more data are needed to clarify this. Certainly, variation in reported LPA levels and subtypes differ between studies, and this is likely due to variations in the LPA subtypes measured or differences in methods used for measurement.

The physiological effects of LPA are mediated through six recognized receptors, LPA 1–6. When referring to human protein they are referred to as LPA1–LPA6 and the corresponding genes as *LPAR1–LPAR6.* Non-human genes are referred to as *Lpar1–Lpar6* [49]. The expression of LPA receptor 1, 3 and 6 mRNA was detected in human HCC tissue [33,44,50], and LPA6 mRNA levels were significantly increased in HCC compared with normal human liver or adjacent non-tumor liver tissue [50]. Another study showed that LPA6 mRNA levels were the most abundant subtype in HCC, and were higher in HCC tissue with microvascular invasion compared to tissues without microvascular invasion [33]. Therefore, while data are limited, LPA receptor expression appears to be increased in the setting of HCC, providing one potential mechanism of increased LPA signaling in this setting.

## 4. Immune Activation and Morbidity in the Setting of Chronic Viral Infection and Aging

Increased systemic inflammation is a hallmark of aging and chronic viral infections such as HIV and HCV. Chronic systemic inflammation and plasma levels of cytokines and soluble receptors released upon cellular activation are associated with immune dysfunction, morbidity, and mortality [51,52,53,54,55] (Table 2). Even HIV-infected patients that have successfully controlled viral replication on antiretroviral therapy (ART) for decades have increased mortality and morbidity and persistent inflammation. Many of the morbidities in these populations are associated with soluble markers of inflammation. For example, IL-6 is one of the most prominent cytokines associated with morbidity and mortality in the elderly [54,56,57] and in chronic HIV disease [52,53]. Although IL-6 may be elevated in HCV patients with advanced liver disease [23,58] it is not always elevated in HCV infection [23,58] and has not yet been associated with morbidity and mortality during HCV infection. Plasma levels of soluble CD14 (sCD14) and soluble CD163 (sCD163) are indicators of monocyte, macrophage or Kupffer cell activation [59,60]. Elevated plasma levels of sCD14 are associated with cardiovascular disease (CVD) and mortality in the elderly [61] and are associated with hepatic inflammation and progression of liver disease in HCV infected patients [58]. Although IL-6 and sCD14 may be produced independently, we have shown that IL-6 can induce production of sCD14 in human PBMC cultures [60], providing proof of concept for a direct causal relation between these markers. Perhaps even more pronounced is the association of the soluble hemoglobin scavenger receptor, sCD163, with liver fibrosis [62,63] and hepatic damage [64,65] in HBV and HCV infected patients. Elevated plasma sCD163 has also been negatively associated with peripheral blood CD4 counts and positively associated with monocyte-platelet aggregates in untreated HIV disease [66]. Additionally, monocyte-platelet aggregates are elevated in CVD [67]. Another study found that sCD163 was elevated in plasma and was associated with non-calcified coronary plaque in HIV-infected patients [68]. Lastly, plasma levels of the inflammatory chemokine, interferon gamma induced protein 10 (IP10) or chemokine interferon gamma inducible protein 10kDa (CXCL10) are elevated in HCV-infected patients [69,70] and are both elevated and associated with rapid disease progression in HIV infection [71,72]. How and if these soluble factors are involved in the pathology of liver damage and what impact they have on long term immune function is yet to be determined. 

As mentioned above, there are numerous studies demonstrating an association between inflammation and mortality and morbidities. Demonstrating a causative relation is more challenging. In the US, heart disease is the leading cause of death (CDC, 2017) and a number of studies have demonstrated a causal role for inflammation. One study in rats found that subcutaneous injection of IL-6 led to deterioration of myocardial contractile function [73] and another study using IL-1 receptor antagonist deficient mice (IL-1Ra−/−) showed that IL-1Ra−/− mice had increased neointima formation after arterial injury [74]. They concluded that IL-1β likely plays a role in restenosis after angioplasty, the development of atherosclerosis, and the metabolism of cholesterol [74]. Several studies in human cohorts have found associations of systemic inflammation and CVD [68,75,76]. Two studies found an association of sCD163 with CVD in treated, HIV-infected men. One found that plasma levels of sCD163 were independently associated with non-calcified coronary plaques in both treated HIV-infected and HIV-seronegative men [68]. The other study found that elevated levels of sCD163, sCD14, and CCL2 (monocyte chemoattractant protein 1) were associated with greater prevalence of coronary artery stenosis. Higher plasma levels of sCD163 specifically, were associated with a greater prevalence of coronary artery calcium, mixed plaque, and calcified plaque in treated HIV-infected men [75]. A human study examining the pathogenic role of IL-6 in CVD found that higher levels of IL-6 were independently associated with reduced systolic function in the septum and inferior wall of the heart [76]. Lastly, a large, randomized, double-blind trial of the anti-IL-1b monoclonal antibody, canakinumab found that blocking the effects of IL-1b with canakinumab led to a significantly lower rate of recurrent cardiovascular events as compared to placebo. This was independent of lipid-level lowering [77].

How does immune activation affect immune function? Poor immune responses to new or previously “unseen” pathogens and poor vaccine response is apparent in the elderly, and in patients with chronic viral infections [78,79,80,81]. We found that systemic inflammation pre-vaccine is associated with poor vaccine response during HCV infection [70]. High plasma levels of IL-6 were negatively associated with anti-HBV IgG antibody titers, and sCD14 and sCD163 were negatively associated with anti-HBV and anti-HAV antibody titers after HAV/HBV neoantigen vaccine in HCV-infected patients [70]. Again, these were associations and the mechanisms remain unclear. However, chronic inflammation may lead to T cell exhaustion/senescence [82,83]. Cells that are continually stimulated to divide eventually become refractory to further stimulation and enter a state of replicative silence referred to as senescence. Proportions of exhausted/senescent T cells are elevated in the elderly [84,85] and in patients with chronic viral infections [86,87,88]. Several studies have suggested that T cell exhaustion/senescence is linked to poor vaccine response during HCV infection [89,90] and in the elderly [91]. 

The ATX/LPA axis and LPA signaling has been illustrated in chronic inflammatory diseases such as idiopathic pulmonary fibrosis, chronic interstitial lung disease, and rheumatoid arthritis [92]. Notably, ATX-LPA signaling may occur in a number of compartments, including the vessel wall, the cell surface as well as intracellularly. In this regard, it has been shown that ATX originating from adipose tissue together with ATX secreted from endothelial cells [93], smooth muscle cells [94] and macrophages [95] can bind to activated β1 and β3 cell integrins [96], resulting in increased catalytic activity and local LPA production [96]. Additionally, LPA can be produced locally in atherosclerotic lesions [97] and it has been shown that LPA can enhance uptake of oxidized phospholipids by macrophages [98]. LPA can modulate monocyte recruitment and activation [99,100], production of reactive oxygen species and release of arachidonic acid and IL-1β [101]. LPA can induce differentiation of monocytes into macrophages as well [102]. We have shown that classic CD14++CD16− monocytes express higher levels of CD86 and CD80 after stimulation with LPA [23]. Finally, LPA also serves as an endogenous toll-like receptor 4 ligand to activate NF-κB signaling. NF-κB signaling has been shown to contribute to development of atherosclerosis and the formation of unstable plaques through enhanced inflammatory cytokine production and matrix metallopeptidase 9 (MMP-9) expression [103]. These ATX/LPA axis effects may, in part, contribute to development of cardiovascular disease, especially atherosclerosis. 

## 5. Elevated Levels of ATX during Chronic HCV Infection Are Associated with Systemic Immune Activation

Chronic hepatitis C virus infection is the most common cause of cirrhosis, end-stage liver disease, and hepatocellular carcinoma in the United States [91,104]. Sustained inflammation and related fibrogenesis represent the basis of liver damage during chronic HCV infection [105,106]. This is characterized by a network of inflammatory chemokines and mediators of innate and adaptive immune system activation. Immunophenotyping of lymphocytes that infiltrate the liver during HCV infection indicates the presence of natural killer (NK) cells, natural killer T (NKT) cells, regulatory T cells, monocytes/macrophages, dendritic cells (DC) and T cells (CD4+, and CD8+), implicating these cellular types in the pathogenesis of disease [107]. Additionally soluble inflammatory markers such as sCD14, IL-6, sCD163, and Mac2BP were associated with liver fibrosis and elevated transaminase levels [58,62]. ATX is one of the markers that has been shown to be elevated in patients with chronic HCV infection [23,29,46]. Sites of chronic inflammation can develop high endothelial venule-like vessels that can serve as a gateway for lymphocyte entry and ATX is expressed and released from lymphoid organ high endothelial venules (HEV) [3,108]. Additionally, ATX is capable of binding to receptors on chemokine activated human lymphocytes in a β1 integrin-dependent manner [3] and may promote lymphocyte migration into inflamed non-lymphoid tissues. As discussed above, ATX can be produced locally by hepatocytes and this appears to contribute to elevated ATX levels observed in persons with HCC, especially those with underlying cirrhosis or HCV infection. In these ways, ATX may contribute to liver inflammation. Additionally, ATX induction within the inflamed tissues may stimulate local LPA production and the downstream effects of LPA signaling. LPA has a variety of effects on lymphocytes, including promoting T-cell proliferation, preventing T-cell death, and inducing inflammatory cytokine expression [109,110]. LPA modulates monocyte migration via induced endothelial cell secretion of interleukin 8 and monocyte chemoattractant protein 1 [111] and by upregulation of endothelial adhesion molecule expression [112]. As mentioned above, LPA can induce monocyte transformation into macrophages with higher expression of CD68 as seen in M1 macrophages [102]. It has been shown that LPA may also affect proliferation of hepatocytes and hepatic stellate cells [18,21]. 

In a discovery data set, we identified elevation of ATX in patients infected with HCV, especially those with indicators of advanced liver damage (high AST to Platelet Ratio Index (APRI) score) [113]. These findings were confirmed in a follow up study, and findings were extended to show increased ATX plasma levels in patients with HCV-HIV co-infection when compared with uninfected controls [23]. We also observed that ATX levels positively correlated with AST and APRI and negatively correlated with PLT counts in HCV infected and HCV-HIV co-infected patients [23]. These findings have been corroborated by three other groups [27,29,46]. 

**Figure 1:**
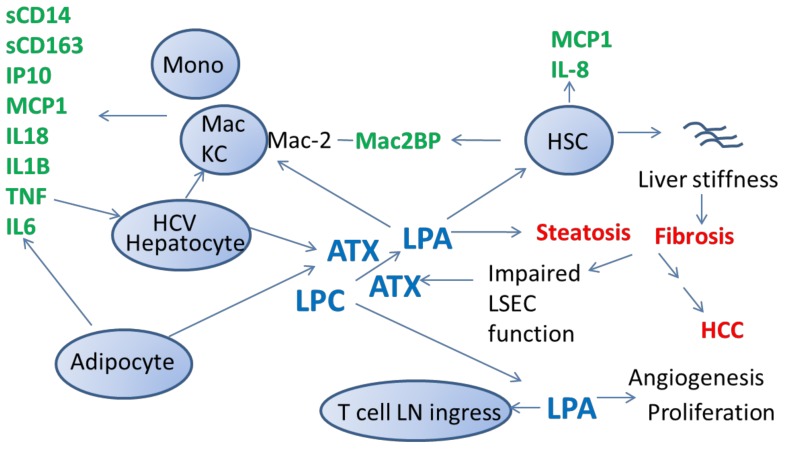
ATX-LPA (lysophosphatidic acid) axis in hepatitis C virus (HCV) pathogenesis. Liver fibrosis and hepatocellular carcinoma outcomes of chronic HCV infection are proposed here to, in part, involve ATX-LPA axis signaling. Initiators in this model include direct HCV action on hepatocytes. ATX-LPA signaling is proposed to be enhanced through augmentation of ATX levels (hepatic synthesis, impaired clearance due to impaired Liver Sinusoidal Endothelial Cell (LSEC) function, and potentially through altered adipocyte and lipid homeostasis), resulting in generation of lysophasphatidic acid (LPA) from lysophosphatidil choline (LPC). Consequences of augmented ATX-LPA signaling likely involve Hepatic Stellate Cell (HSC) activation, monocyte (Mono), macrophage (Mac), and Kupffer cell (KC) activation, angiogenesis, proliferation, and elaboration of soluble factors such as sCD14, sCD163, IL-6, IL-1b, TNF, IL-18, MCP1, Mac2BP, IP10, which result in immunocyte tissue localization, inflammation and fibrosis. Interleukin (IL); interferon gamma induced protein 10 (IP10); monocyte chemoattractant protein 1 (MCP1); tumor necrosis factor alpha (TNFa)

Because LPA is the enzymatic byproduct of ATX activity [4], and because LPA is a known lipid mediator of cellular immune activation [16], it is reasonable to consider the ATX-LPA axis as a mediator of systemic immune activation. In this regard, we have observed that ATX plasma levels correlate with LPA levels, IL-6, sCD14, sCD163 and Mac2BP levels in patients with chronic HCV infection [23]. Specific mechanisms linking the ATX-LPA axis to inflammation and pathogenesis of HCV associated disease, including liver fibrosis and HCC, are yet to be clarified. However, it appears likely that a complex state of chronic immune activation includes monocytes, macrophages, Kupffer cells, T cells, NK cells, and Hepatic Stellate Cells. Soluble factors likely include IL-1β, IL-6, IP-10, and tumor necrosis factor alpha (TNFα) [23,114,115,116]. Pathways such as ATX-LPA, hepatocyte apoptosis and oxidative stress are likely engaged. We have outlined one potential model linking these factors based upon the literature (Figure 1). 

## 6. Partial Normalization of ATX Levels within Months of Starting IFN-Free DAA HCV Therapy Is Associated with Variable Degrees of Normalization of Parameters of Systemic Immune Activation

We have observed a partial normalization of ATX levels within months of starting IFN-free direct acting antiviral (DAA) HCV therapy, consistent with a non-fibrotic liver disease component contributing to elevated ATX levels during chronic HCV infection. Furthermore, partial normalization of ATX levels were accompanied by partial normalization of plasma LPA species levels, and ATX levels correlated with LPA levels [23]. Importantly, partial normalization of ATX levels during HCV DAA therapy has been verified by others in larger patient cohorts [117]. Notably, ATX plasma levels in HCV-infected patients declined more so in those patients that achieved a response to therapy compared to those that did not respond to therapy [117].

Concordant with partial normalization of ATX levels, we have observed partial and variable normalization of parameters of systemic immune activation during DAA therapy for HCV [23]. In particular, levels of sCD163 and Mac2BP partially normalize, while levels of IL-6 do not change and levels of sCD14 do not consistently change within 24 weeks of starting HCV DAA therapy [23,26]. Furthermore, relationships between levels of ATX and markers of immune activation present prior to therapy onset are often no longer present after therapy completion. These observations support a model where causal linkages exist between HCV, ATX, liver damage/fibrosis and systemic immune activation.

## 7. Concluding Remarks

- ATX is elevated during a number of chronic liver disease states, most commonly associating with degree of liver fibrosis. Levels of ATX are most commonly associated with levels of the ATX enzymatic activity product, LPA, a lipid mediator that signals through LPA receptors.
- The ATX-LPA axis is associated with a number of biologic pathways including airway inflammation, wound healing, as well as a variety of cancers, and immune modulation. In regard to the latter, the ATX-LPA axis is associated with systemic immune activation in the settings of HCV and HCV/HIV infection.
- Since systemic immune activation is associated with morbidity and mortality during chronic viral infection, and since ATX-LPA signaling may contribute to immune activation, further investigation of the relationship between ATX-LPA signaling and morbidity during chronic viral infection is warranted.
- ATX plasma levels and markers of systemic immune activation partially normalize with HCV direct-acting antiviral therapy, indicating a non-fibrotic and reversible component contributing to elevated ATX levels during chronic hepatic viral infection.

## Figures and Tables

**Table 1:** Liver diseases associated with elevated autotaxin (ATX) levels.

Study	Disease	ATX Level/Activity	Method of Measurement
Joshita et al. [25] Wunsch et al. [24]	Primary biliary cirrhosis	0.97 mg/L (0.79–1.11) (serum) 10.2 ± 4.4 nmol/ml/min	2-site enzyme immunoassay Activity assay method
Dhillon et al. [30] Wunsch et al 2016 [24]	Primary Sclerosing Cholangitis	6.3 nmol/ml/min 7.3 ± 3.4 nmol ml min	Activity assay method Activity assay method
Yamazaki et al. [29] Kostadinova et al. [23] Pleli et al. [27]	Chronic HCV infection	1.39 (1.01–1.99) mg/L (serum) 0.77 mg/L (plasma) 0.814 ± 0.42 mg/L (serum)	2-site enzyme immunoassay, ELISA ELISA
Joshita et al. [22]	Chronic HBV infection	1.22 mg/L (serum)	2-site enzyme immunoassay
Fujimori et al. [22] Honda et al. [31] Rachakonda et al. [28]	NAFLD^1^	0.86 mg/L (serum) 0.298 mg/L (serum) 0.374 mg/l (serum)	2-site enzyme immunoassay 2-site enzyme immunoassay ELISA
Kondo et al. [32] Enooku et al. [33]	Hepatocellular carcinoma (HCC)	2.21 ± 1.03 mg/L serum 1.068 mg/L serum	2-site enzyme immunoassay 2-site enzyme immunoassay
Nakamura et al. [34] Pleli et al. [27] Fujimori et al. [22] Kostadinova et al. [23] Wunsch et al [24]	Healthy controls	0.731 ± 0.176 mg/L serum 0.258 ± 0.40 mg/L (serum) 0.76 mg/L (serum) 0.4 mg/L (plasma) 2.8 ± 1.4 nmol/ml/min	2-site enzyme immunoassay ELISA 2site enzyme immunoassay ELISA Activity assay method

^1^NAFLD = non-alcoholic fatty liver disease.

**Table 2:** Cytokine/soluble receptor and associated morbidities.

Cytokine or Soluble Receptor	Patient Group	Morbidity	Mortality	Reference
IL-6	Elderly		X	[54,57]
	Elderly	Osteoporosis, Alzheimer’s disease, neoplasia, frailty		[56]
	HIV		X	[52]
	HIV	Non-AIDS-defining events: myocardial infarction, stroke, malignancies, serious bacterial infection		[53]
sCD14	Elderly	CVD (carotid wall thickness, ankle-brachial index)	X	[61]
	HCV	Hepatic inflammation, liver fibrosis		[58]
sCD163	HCV	Liver fibrosis		[62,63]
	HCV/HIV coinfection	Hepatic damage (AST, ALT)		[65]
	HCV/HIV coinfection	Hepatic fibrosis (necroinflammation, Ishak fibrosis score, non-invasive fibrous score)		[64]
	HIV	Non-calcified coronary plaque		[68]
